# Vesico-appendiceal fistula secondary to adenocarcinoma of the appendix: a case report and literature review

**DOI:** 10.11604/pamj.2020.37.97.10655

**Published:** 2020-09-27

**Authors:** Adriá Rosat, Eduardo Pérez, Juan Manuel Sánchez, Omar Bilal Halawa González, Manuel Barrera

**Affiliations:** 1 Department of General Surgery, Hospital Universitario Nuestra Señora de Candelaria, Cruz de Tenerife, Spain,; 2 Urology Service, Hospital Universitario Nuestra Señora de Candelaria, Cruz de Tenerife, Spain,; 3 Transplantation Surgery Unit and General Surgery Service, Hospital Universitario Nuestra Señora de Candelaria, Cruz de Tenerife, Spain

**Keywords:** Vesico-appendiceal fistula, adenocarcinoma appendix, literature review

## Abstract

A 50-year-old woman presented with a 5-month history of recurrent urinary tract infections. She had no complaints of any intestinal symptoms. She had been treated previously with oral antibiotics. The episodes became more frequent and she started with pain in the lower abdomen and fetid urine. Complete study lead to diagnosis of adenocarcinoma of the appendix with bladder fistula. The lesion was removed by laparoscopic right hemicolectomy and en bloc partial cystectomy. Pathological examination revealed a mucinous adenocarcinoma that had originated in the appendix and extended into the bladder wall. Six years after the operation, the patient remains asymptomatic with no evidence of recurrent or metastatic disease. Appendiceal carcinoma extending to the bladder is extremely rare and approximately 40 cases have been described. Management of recurrent urinary tract infections should not limit to empiric antibiotic therapy before the exclusion of possible organic causes. Appendiceal carcinoma may invade the bladder without intestinal symptoms but with urinary symptoms only, because of its anatomical position. The recommended treatment for non-carcinoid appendiceal tumours is right hemicolectomy and for T4 tumours en bloc resection of the involved structures. Further study is needed to determine adjuvant therapy. A literature review was made.

## Introduction

It is well known that tumors or infection of the adjacent organs, such as the rectum or the sigmoid colon, can invade or infiltrate the bladder. Of these cases, adenocarcinoma of the appendix invading the urinary bladder is very rare and it is very difficult to make a correct diagnosis preoperatively. We describe a case of adenocarcinoma of the appendix who presented with a vesico-appendiceal fistula.

## Patient and observation

**Figure 1:**
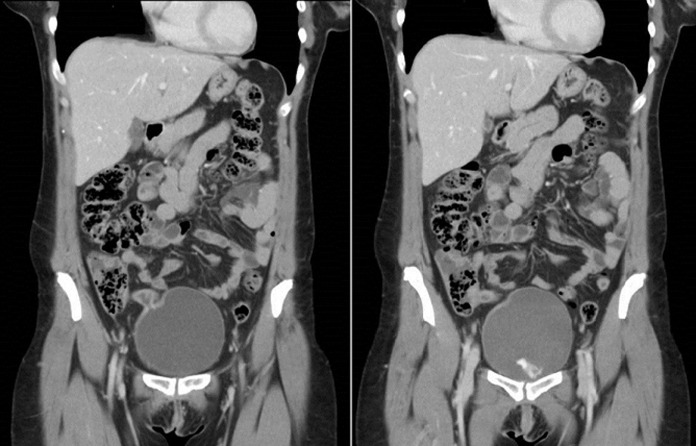
coronal multiplanar reconstruction (MPR) of the computed tomography images showing the fistula between the ileocecal junction and the bladder, pointed by arrows

**Figure 2:**
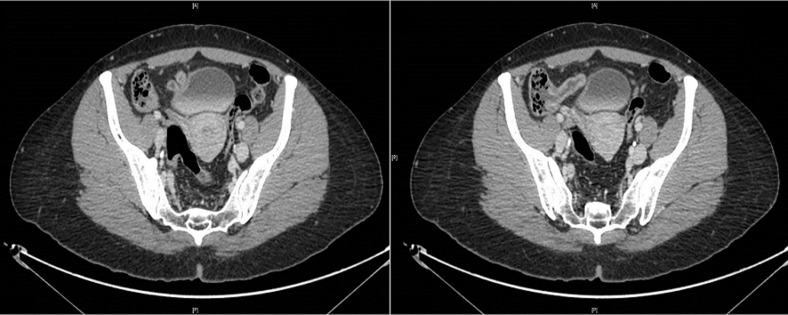
enhanced abdominal computed tomography scan (transverse images), showing the fistula between the ileocecal junction and the bladder, pointed by arrows

A 50-year-old woman presented with a 5-month history of urinary frequency and micturition pain. She had no complaints of any intestinal symptoms such as pain, obstruction or melena. She had been treated previously with oral antibiotics for recurrent urinary tract infections. These became more frequent and she started with pain in the lower abdomen and fetid urine. Ultrasonography found a nodular image in the right posterior wall of the bladder. Cystoscopy confirmed the edematous broad-based tumorous lesion in the right posterior wall of the bladder. Transurethral biopsy indicated the presence of a mucinous adenocarcinoma of probable colonic origin. Endoscopy showed a protrusion in the cecum with origin in the appendix and mucoid content. Pelvic enhanced computed tomography (CT) revealed circumferential thickening of the appendix with mucoid material inside and no metastases. The point of the appendix was invading the muscle layer of the bladder, which was thickened (Figure 1, Figure 2). With the diagnosis of adenocarcinoma of the appendix and bladder fistula we performed a programmed surgery. The lesion was removed by laparoscopic right hemicolectomy and en bloc partial cystectomy. Pathological examination revealed a mucinous adenocarcinoma that had originated in the appendix and extended into the bladder wall. There was no evidence of lymphatic spread in 16 lymph nodes examined (pT4N0M0). In our case the laparoscopic approach allowed a short hospitalization and patient was discharged home on 5^th^ postoperative day. She received adjuvant chemotherapy with 5-fluorouracil and oxalyplatinum. Six years after the operation, the patient remains asymptomatic with no evidence of recurrent or metastatic disease.

## Discussion

A vesicoenteric fistula is usually a complication of an inflammatory or neoplastic process. Common causes of fistula formation include colonic diverticulitis (51% of the cases), colorectal adenocarcinoma (16%), Crohn's disease (12%) and bladder carcinoma (5%) [1]. Vesico-appendiceal fistulas are rare, constituting less than 5% of all vesicoenteric fistulas [2]. The most common cause of a vesico-appendiceal fistula is the failure to recognize and treat acute appendicitis. Such fistulas occur most commonly in patients between 10 and 40 years old [2]. Appendiceal adenocarcinoma is rare with a frequency of 0.08% of all appendices removed surgically [3]. The condition was first described by Berger in 1882 [4]. Ferro and Anthony reported approximately 250 cases of adenocarcinoma of the appendix. This disorder comprises 0.2 to 0.5% of all gastrointestinal neoplasms [5]. Moreover, appendiceal carcinoma extending to the bladder is extremely rare and approximately 40 cases have been reported [6-16]. Kimura *et al*. gathered a series of 21 cases in Japan [16]. The main lymphatic and venous drainage of the appendix is essentially that of the cecum and terminal ileum. Metastasis occurs via lymphatics and the initial spread of the tumor is to the ileocolonic nodal basin as well as the infraduodenal and para-aortic areas. Therefore, the recommended treatment for non-carcinoid appendiceal tumours is right hemicolectomy and for T4 tumours en bloc resection of the involved structures [17], as performed in most of the reported cases. It has a 5-year survival rate of 68% in contrast to 20% 5-year survival rate after appendectomy alone [18]. The present case received adjuvant chemotherapy with 5-fluorouracil and oxalyplatinum.

Early diagnosis of carcinoma of the appendix is difficult because of the absence of specific symptoms. This is because of its anatomical position, which does not interfere with the intestinal passage in its early stage. Therefore, appendiceal carcinoma is always neglected or misdiagnosed. Clinical presentations include acute appendicitis, abdominal mass or intestinal obstruction. In two studies, none of the patients were correctly diagnosed preoperatively [18,19] and 45% had widespread abdominal carcinomatosis at presentation. The distinction between an appendiceal carcinoma and appendicitis is difficult preoperatively, but appendiceal carcinoma should be suspected when patients present with a longer duration of symptoms [20]. In some patients transurethral biopsy revealed adenocarcinoma and the tumor was supposed to invade from the colon. The radiologic findings on computed tomography, magnetic resonance imaging or barium enemas are variable and include extrinsic compression or enterovesical fistula formation, depending on the tumor size and position. Therefore, a diagnosis of a primary appendiceal mucinous carcinoma must be considered by radiologists and clinicians for patients who do not exhibit gastrointestinal symptoms, but show involvement of the nearest organs and the bladder wall. It is possible that computed tomography multiplanar reconstruction images may provide more information.

## Conclusion

Management of recurrent urinary tract infections should not limit to empiric antibiotic therapy before the exclusion of possible organic causes. Appendiceal carcinoma may invade the bladder without intestinal symptoms but with urinary symptoms only, because of its anatomical position. The recommended treatment for non-carcinoid appendiceal tumours is right hemicolectomy and for T4 tumours en bloc resection of the involved structures. Further study is needed to determine adjuvant therapy.

**Consent:** patient´s written consent was obtained and is available upon request.
